# Supplementary material for: Wearable Online Freezing of Gait Detection and Cueing System
Source: Bioengineering (Basel). 2024 Oct 20;11(10):1048. doi: 10.3390/bioengineering11101048 (PMC11505507; doi:10.3390/bioengineering11101048)
Supplement: Supplementary file 1 [file bioengineering-11-01048-s001.zip › Supplementary S1.pdf]

1. CNN+RNN+PS algorithm parameters

Classification algorithm	Algorithm parameters for CWT dataset	Algorithm parameters for RAW dataset
CNN+RNN+PS	Num. Learnable parameters = 125k,<br>Optimisator = 'ADAM',<br>Batch size = 8192,<br>PS set = [1],<br>Shuffle = 'every iteration',<br>Num iterations = 100,<br>Learning rate (LR) = 0,01,<br>Loss function = binary cross entropy,<br>Model's output = best validation loss,<br>Inpuput dimensionality: 540 x 1,<br>Output dimensionality: 1 x 1.<br><br>*Layers (for PS = 1):*<br>*Input layer (540);*<br>*Reshaping layer (1 x 540)*<br>*Batch normalization;*<br>*1D convolution (filt.Size=2; num.filt.=64);*<br>*Relu layer;*<br>*1D convolution (filt.Size=2; num.filt.=64);*<br>*Relu layer;*<br>*1D convolution (filt.Size=2; num.filt.=64);*<br>*Relu layer;*<br>*Flatten layer;*<br>*Fully connected layer (64);*<br>*Relu layer;*<br>*Fully connected layer (5);*<br>*Softmax layer;*	Num. Learnable parameters = 124k,<br>Optimisator = 'ADAM',<br>Batch size = 8192,<br>PS set = [10, 15, 20, 30, 40, 50, 60, 70, 80, 100],<br>Shuffle = 'every iteration',<br>Num iterations = 100,<br>Learning rate (LR) = 0,01,<br>Loss function = binary cross entropy,<br>Model's output = best validation loss,<br>Input dimensions: 800 x 1 (Za PS = 40),<br>Output dimensions: 1 x 1.<br><br>*Layers (for PS = 40):*<br>*Input layer (800);*<br>*Reshaping layer (40 x 20)*<br>*Batch normalization;*<br>*1D convolution (filt.Size=2; num.filt.=64);*<br>*Relu layer;*<br>*1D convolution (filt.Size=2; num.filt.=64);*<br>*Relu layer;*<br>*1D convolution (filt.Size=2; num.filt.=64);*<br>*Relu layer;*<br>*Ravnalni layer;*<br>*Fully connected layer (64);*<br>*Relu layer;*<br>*Fully connected layer (5);*<br>*Softmax layer;*

2. CNN+RNN+PS algorithm initialization pseudocode

```
Definition of input:
inputs = input layer(20 * past_samples)

Reshaping and normalization:
x = Reshape(inputs into shape (20, past_samples))(inputs)
x = Batch Normalization(x)

Adding convolutional layers:
x = 1D convolutional layer (x, filters=64, activation='relu')(x)
x = 1D convolutional layer (x, filters=64, activation='relu')(x)
x = 1D convolutional layer (x, filters=64, activation='relu')(x)

Final processing layers:
x = Flatten layer(x)
x = Fully connected layer (x, 64, 'relu')(x)

Definition of output:
outputs = Fully connected layer (x, 1, 'sigmoid')(x)

Define the complete model:
model = Create model(inputs, outputs)
```

3. CNN+RNN+PS algorithm learning pseudocode

Definition of the set of past samples:
past_samples = [10, 15, 20, 30, 40, 50, 60, 70, 80, 100]

Data preprocessing:
For each sample in 'past_samples':
Generate training and testing data (X_train, X_test, Y_train, Y_test)

Model training:
Set the number of independent model training runs (iterations)
Set a variable to store the best result (best_score)

For each sample in 'past_samples':
For i from 1 to iterations:
Create and compile the model: model = build_model()
Train the model: model.fit(X_train, Y_train)
Evaluate the model: score = model.evaluate(X_test, Y_test)

If score > best_score:
best_score = score
Save the model: model.save('model_best.h5')

4. Pseudocode for converting Keras model into C code

Create a TFLite converter from the Keras model
Convert the model to TFLite format
Open a file and write the TFLite model into it

Function: Convert hex values to a C array:

Define a function with name and hex data as parameters
Initialize an empty string for the C code

Add header guard:

Add '#ifndef' with the variable name and '_H'
Add '#define' with the variable name and '_H'

Add the table length at the top of the file
Declare a C variable as an array

For each hex data:

Format a string from the hex values
Add a comma if it's not the last element
Add a new line every 12 elements
Add the hex string to the array

Add a closing brace
Add '#endif' with the variable name and '_H'

Return the C code string

Write the TFLite model to a C file:

Open the file for writing
Write the C code generated by the function to the file
Close the file
